# Investigation of Friction and Erosion Wear Properties of Titanium and Titanium Alloy Pipes

**DOI:** 10.3390/ma17205043

**Published:** 2024-10-15

**Authors:** Ting Mao, Zhiming Yu, Jing Yan, Yong Xu, Shibo Zhang, Lincai Peng

**Affiliations:** 1 Research Institute of Natural Gas Technology, PetroChina Southwest Oil and Gasfield Company, Chengdu 610213, China; 2 National Energy R&D Center for High Sulfur Gas Reservoir Exploitation, Chengdu 610213, China; 3 Pilot Test Base for High Sulfur Gas Reservoir Exploitation of CNPC, Chengdu 610213, China; 4 Southwest Oil and Gas Field Company, Chengdu 610051, China; 5 Chengdu Tianfu New District Huatian Star Energy Gas of Sichuan Huayou Group Co., Ltd., Chengdu 610213, China

**Keywords:** commercially pure titanium TA3, titanium alloy, friction and wear, erosion wear, gathering pipeline

## Abstract

Titanium alloys are applied in oil and gas development and transportation to improve conditions because of their high specific strength and corrosion resistance. However, the disadvantage of poor wear resistance has become an obstacle to developing titanium alloys. The friction and wear properties of pure titanium TA3 and titanium alloy TA10 were tested under different loads and different friction forms using a reciprocating friction and wear tester. Moreover, the erosion resistance of pure titanium TA3 and titanium alloy TA10 was studied using a gas–solid erosion tester. The results show that the wear rate of TA3 and titanium alloy TA10 increases with increasing friction load. Under a load of 50 N, the mass losses of TA3 under dry friction and wet friction were 0.0013 g and 0.0045 g, respectively, while the mass losses of TA10 were 0.0033 g and 0.0046 g, respectively. While the load increased to 70 N, the mass loss of TA3 was even greater, reaching 0.0065 g, and the mass loss of TA10 was 0.0058 g. The wear forms of TA3 and TA10 include abrasive wear, adhesive wear and oxidation wear. The joint action of various friction forms leads to the loss of materials. Under the simulated working conditions, the erosion rates of TA3 and TA10 were 1.01 × 10^−3^ g/s and 0.94 × 10^−3^ g/s. The erosion mechanism is the same, including plowing, indentation and cracking.

## 1. Introduction

For high-temperature, high-pressure and high-sulfuric acid oil and gas wells, the reservoir fluid usually contains a high concentration of H_2_S\CO_2_ and a high concentration of Cl^−^ [1]. At the same time, deepwater and ultra-deepwater oil and gas exploitation in complex marine environments has higher requirements for corrosion resistance and the high stability of oil and gas pipelines [2]. Based on these complex and harsh service conditions, the conventional materials of carbon steel, low alloy steel and stainless steel can no longer meet the requirements. With improvements in material selection and research at home and abroad, the titanium alloy has attracted significant attention because of its high strength ratio and high corrosion resistance. It has become the preferred material for oil and gas exploitation and transportation under adverse working conditions, such as high sulfuric acid content and complex deep-water oceans [3,4,5].

The United States and Japan first began to study the applicability of titanium alloy materials in the field of oil and gas development as early as the mid-1980s [6]. It has been applied in high-pressure and high-temperature wells, surface gathering pipelines and submarine pipelines [7,8,9,10]. The excellent corrosion resistance of titanium alloy lies in the dense and stable oxide film on the surface [10,11,12]. At the same time, this means that once the oxide film on the surface is damaged, the corrosion resistance and wear resistance of the titanium alloy will be affected [13,14]. With the popularization and application of the titanium alloy, it has been found that the titanium alloy has low hardness and poor wear resistance [15,16]. The damage risk to the oil well string surface mainly includes friction wear and erosion wear. On the one hand, the aperiodic and violent vibration of the string induced by the high-speed fluid in the tube will increase the axial load of the string, cause the buckling deformation of the string, and increase the risk of tubing wear failure [17,18]. On the other hand, the flow of gas-liquid-solid multiphase flow and the maintenance of scale and wax removal in the gathering pipeline can easily lead to erosion and friction damage in the pipeline [19], thus reducing the quality of oil transportation and increasing the production cost. Therefore, the friction and erosion wear of titanium alloy materials for oil and gas has become the focus of research that can not be ignored. Friction and wear is a phenomenon wherein the surface material is constantly lost or residual deformation occurs when the friction pair moves relatively. Erosion occurs due to the mechanical interaction between the solid surface and the impacted fluid or solid particles, resulting in the gradual loss of the original material from the solid surface [20,21].

In fact, the friction and wear of titanium alloy have been studied for a long time. The first studies, conducted between 1960 and 1981, showed that titanium alloy had poor wear resistance and put forward a solution to improve the wear resistance of titanium alloy [22,23,24]. Chen et al. [25] studied the effect of oxidation time and sodium tungstate addition on the wear resistance of a micro-arc oxidation layer on a titanium alloy drill pipe; Li et al. [26] studied the friction and wear behavior of vacuum nitriding on a Ti80 titanium alloy; Yao et al. [27,28] successively studied the effect of temperature on the tubing wear properties and friction coefficient of the TC4 titanium alloy. Liu et al. [29] carried out a study on the friction behavior of three kinds of titanium alloy drill pipes under the simulated working conditions of ultra-deep drilling. Thus, it can be seen that the research on titanium alloy oil well string is mostly focused on the technology to improve the surface wear resistance, while the research on the friction and wear behavior and wear mechanism of titanium alloy oil well string is relatively limited. At the same time, these friction and wear studies are mainly focused on drill pipe and casing, and the material is mainly TC4. In the process of gas well production, the air flow is often mixed with cuttings and sand produced by the formation, which may cause erosion in the gathering pipeline [30]. Previous studies on the erosion of materials in oil and gas wells and pipelines have made some advancements [31,32], but these studies are mainly regarding liquid–solid erosion, and most studies use carbon steel and stainless steel as the research materials. At the same time, because of the many factors influencing erosion itself, numerical simulation has become the main means of erosion research [33,34]. However, the experimental study on the gas–solid erosion damage of titanium alloy gathering pipelines is rarely reported.

Among the titanium and titanium alloys used in oil and gas exploitation and production, industrial pure titanium (TA1, TA2 and TA3) and titanium alloy TA10 (Ti-0.3Mo-0.8Ni) are commonly used in seawater pipelines and ground pipelines, respectively [6,35]. Therefore, this paper takes TA3 and TA10 as the research objects, uses the linear reciprocating friction and wear tester to test the friction and wear performance, and analyzes the wear performance under different loads and friction forms. The erosion wear performance is tested using a gas–solid erosion tester under simulated working conditions, and on this basis, the damage mechanism of the friction and erosion wear of TA3 and TA10 is explored in order to provide a reference for the theoretical research and engineering application of friction and erosion of titanium alloys used in oil and gas pipelines.

## 2. Experiments

### 2.1. Characterization of Materials and Microstructure

The materials used are TA3 and TA10, whose chemical composition is shown in Table 1. The hardness values of samples were measured using a HVS-1000 type digital microhardness tester with a load of 1000 g and a hold time of 10 s, and the results are shown in Table 2. The mass losses before and after the wear test and erosion test were weighed by an electronic balance with an accuracy of 0.1 mg. The morphology of wear and erosion was observed and the width and depth of the wear marks were measured by body microscope and laser confocal microscope. The macroscopic and microscopic morphologies of the wear marks were analyzed by scanning electron microscope (JSM7500F, Hitachi, Tokyo, Japan), and the element composition of wear marks was analyzed by energy dispersive spectrometer (INCA X-max50, Oxford Instruments, Oxford, UK). The specific wear rate was calculated and the wear mechanism was explored.

### 2.2. Friction and Wear Performance Test

**Figure 1:**
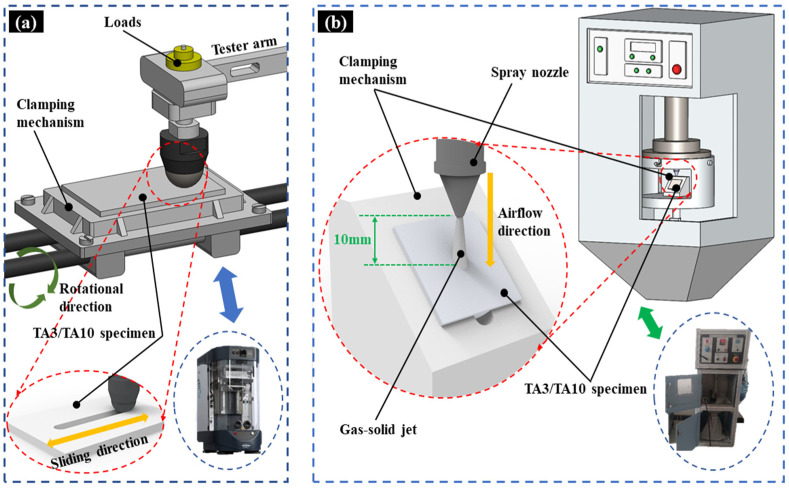
Experimental setup; (**a**) multi-function friction and wear tester, (**b**) air jet erosion tester.

The friction and wear test are carried out using a multi-function friction and wear tester (Bruker UMT-TriboLab, Billerica, MA, USA). The equipment can be used to carry out standard tests and a variety of conventional tribological tests, and the measured data of friction force, load, torque and displacement can be obtained at the same time. The experimental equipment and test dimensions are shown in Figure 1a. According to the ASTM-G133 standard [36], linear reciprocating friction and wear tests were carried out on a ball-to-disk device using a silicon nitride friction ball with a diameter of 10 mm and a hardness of 1500 HV at room temperature. According to on-site monitoring, there is residual liquid inside the pipeline and the friction load range during the pipeline cleaning process is 50 N~70 N. The load size and friction form are set for the test; the load includes 50 N and 70 N and the load form includes dry friction (air friction) and wet friction (3.5%NaCl). The specific experimental scheme is shown in Table 3. Each test was carried out twice, the average value of the test results was taken, and each test was carried out on a new polished sample surface to ensure that the initial conditions of all tests were the same. To facilitate the comparison, this paper uses the corresponding sequence number of each sample to sort and compare.

### 2.3. Gas–Solid Erosion Test

The gas–solid erosion test (Ducom Instruments, New York, NY, USA) was carried out using an air-jet erosion test bed, the air source and pressure were provided by a test machine with a piston air compressor, and a freeze dryer was used to remove moisture from the air flow to ensure the drying of the air flow. According to the ASTM-G76 standard [37], the abrasives used in the test were irregular Al_2_O_3_ angular particles with a median particle size of 50 μm and a Morse hardness of 9. All the tests were carried out at room temperature, and the equipment was calibrated before the test to ensure that its accuracy meets the requirements of the ASTM-G76 standard. In addition, the particle velocity of the device under different pressure settings was measured by the double-circle rotation method, and the accuracy was ±2 m/s. The particle mass flow rate and particle velocity are checked regularly, every 20 tests, to ensure the accuracy and stability of the test conditions. The experimental equipment and sample dimensions are shown in Figure 1b. The gas–solid erosion tests were carried out at room temperature, and the gas–solid erosion tests of TA3 and TA10 were carried out at fixed impact angle, particle impact velocity, particle mass flow rate and erosion time. The specific experimental parameters are shown in Table 4.

## 3. Results

### 3.1. Macroscopic Morphology Analysis

**Figure 2:**
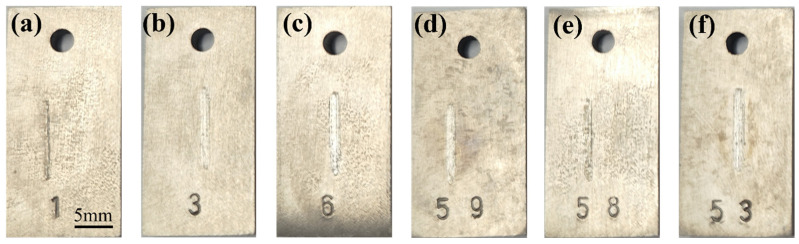
Macroscopic morphology of TA3 and TA10 after friction and wear tests under different working conditions ((**a**): 1#, (**b**): 2#, (**c**): 3#, (**d**): 4#, (**e**): 5#, (**f**): 6#).

**Figure 3:**
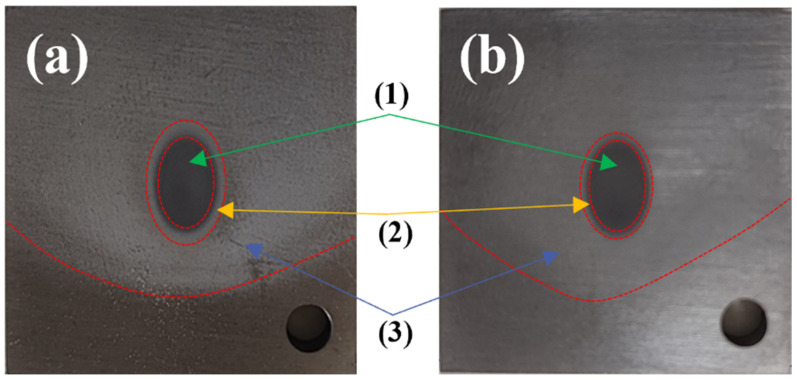
Macroscopic morphology of TA3 and TA10 after gas–solid erosion test ((**a**): TA3 and (**b**): TA10).

Typical samples were selected to observe the macroscopic morphology after the friction and wear test and gas–solid erosion test, as shown in Figure 2 and Figure 3. It can be seen from Figure 2 that the friction load and friction form have a certain influence on the wear resistance of TA3 and TA10. The width of the friction mark is different under different working conditions, and the friction mark of the TA3 sample is the narrowest under the 50 N wet friction condition, while that of the TA3 and TA10 samples is wider under 70 N wet friction condition. In order to make an accurate quantitative analysis of the depth and width of friction marks, confocal microscopy will be used later. As can be seen from Figure 3, the sample can be divided into three parts according to the degree of erosion damage, including (1) the center part of the ellipse with the most severe erosion damage, (2) the darkest color and the part of the ellipse halo with slight and light color erosion damage and (3) the arc part covered with a layer of fine sand outside the halo.

### 3.2. Friction and Wear Properties

#### 3.2.1. Average Friction Coefficient

**Figure 4:**
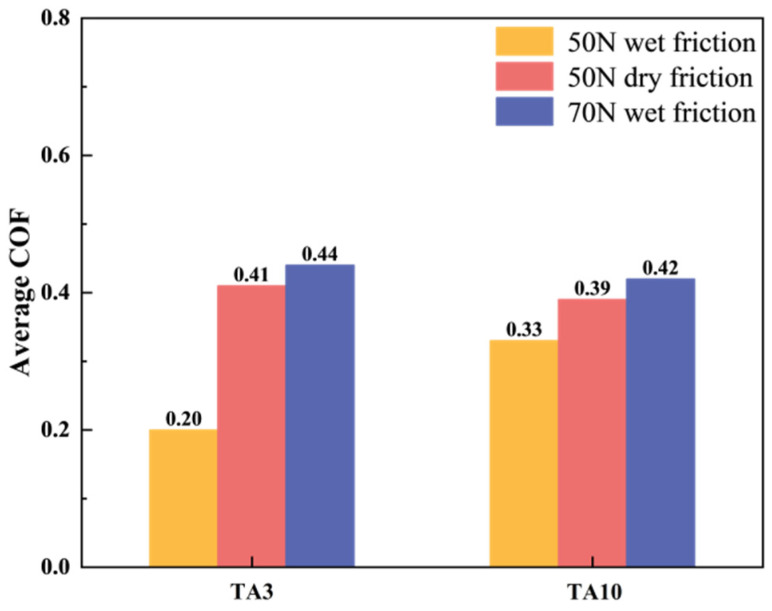
Friction factor of TA3 and TA10 wear specimens.

**Figure 5:**
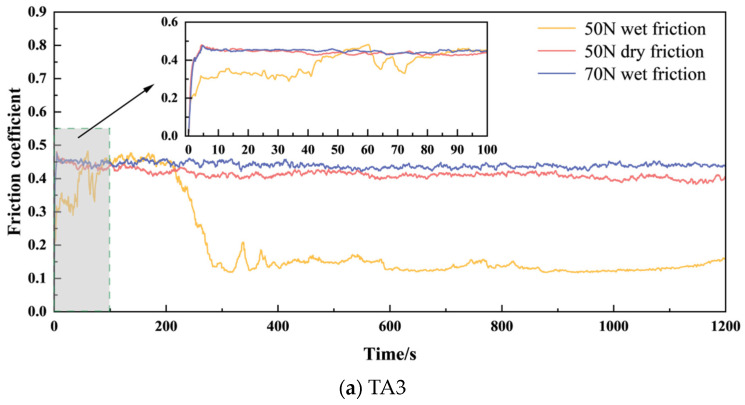

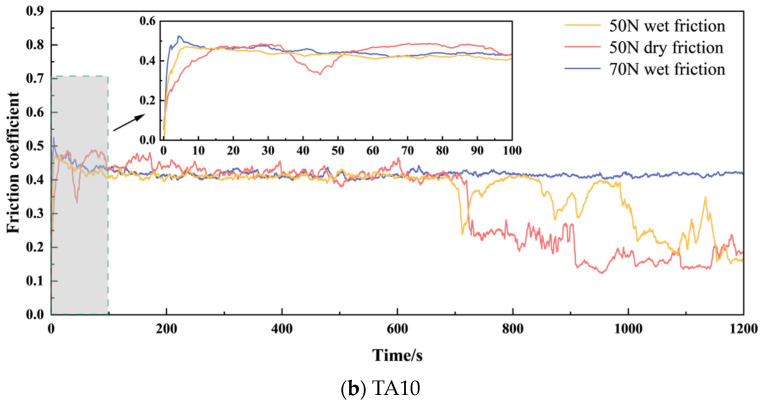
The curve of friction factor with time.

**Figure 6:**
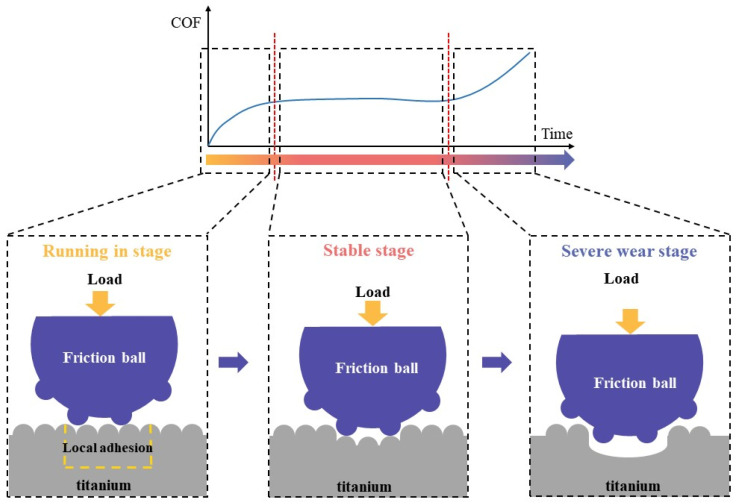
Three stages of surface wear.

The friction factor is one of the important indexes to measure the friction and wear properties of materials. The average friction coefficients of TA3 and TA10 under three working conditions are shown in Figure 4, and the order of the average friction coefficients of the six groups is 3# > 6# > 2# > 5# > 4# > 1#. The average friction coefficient of TA10 under 50 N wet friction is higher than that of TA3, but the average friction coefficient of 50 N dry friction and 70 N wet friction is similar. The variation of friction coefficient of TA3 and TA10 wear specimens with test time under three working conditions is shown in Figure 5. Generally speaking, the friction and wear process can be divided into the running and stable stage and severe friction stage, as shown in Figure 6; the friction process is maintained in the stable stage most of the time, and the initial running and stage time is very short. Under the wet friction condition of 50 N, the friction coefficient of TA3 sample increases to about 0.4 after 0.100 s and maintains this until about 200 s, then begins to decrease below 200 N and 300 s, and finally fluctuates around 0.15 under 50 N dry friction, while the friction coefficient of TA3 specimen under 50 N dry friction is about 0.4 after the rapid increase at the beginning of 5 s. At the same time, a similar phenomenon can be found by comparing the friction coefficient curves of TA10 samples under 50 N wet friction and 50 N dry friction. This shows that during the friction and wear test, the addition of liquid can effectively reduce the friction resistance and friction coefficient. It can be observed from Figure 4 that when the sample material and friction form are the same, the increase in load leads to a significant increase in friction coefficient. When only considering the dry friction condition, it is found that the average friction coefficients of TA3 and TA10 are both larger and close to each other at 70 N. When only considering the wet friction condition, it is found that when the friction load is 50 N, the average friction coefficient of TA3 is about 39% smaller than that of TA10, and when the friction load is 70 N, the average friction coefficient of TA3 and TA10 are very close.

#### 3.2.2. Friction and Wear Morphology

**Figure 7:**
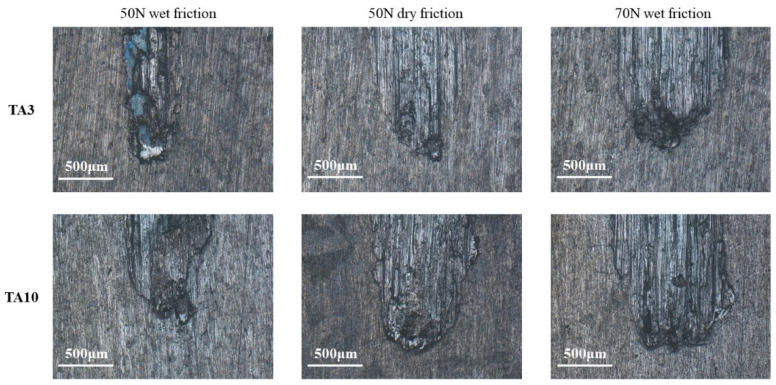
Microscopic images of wear topography under different friction conditions.

The morphology of the TA3 and TA10 wear samples under different friction tests is shown in Figure 7, in which the friction mark width of the TA3 sample under 50 N wet friction conditions is the smallest, and that of the TA3 and TA10 sample under 70 N wet friction conditions is larger. It can be seen from Figure 7 that some of the friction marks on the sample surface are light blue. According to previous studies, it is speculated that a new oxide film is formed when the new metal matrix is exposed to friction. According to observation under the high-power mirror, it can be seen that part of the matrix material of the friction mark fell off during the test, and due to the reciprocating movement of the friction ball, the fallen material accumulated at both ends, resulting in the small width of the friction mark at both ends, which is similar to the ellipse shape.

**Figure 8:**
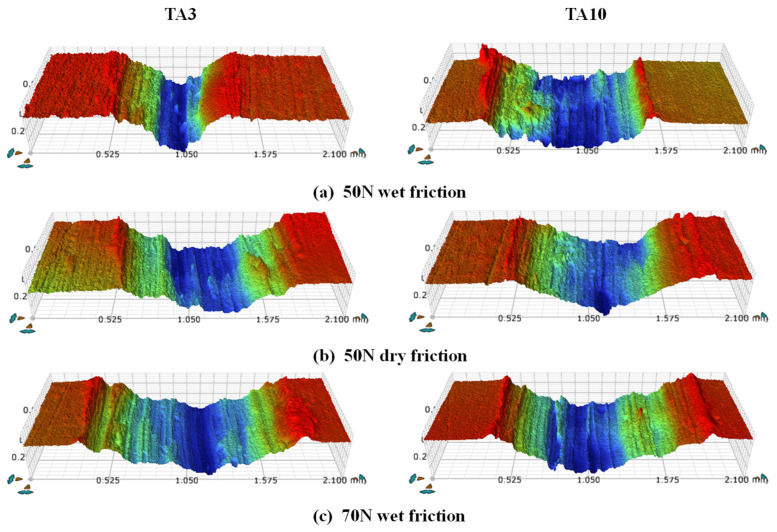
Observation of wear morphology by laser confocal microscope.

**Figure 9:**
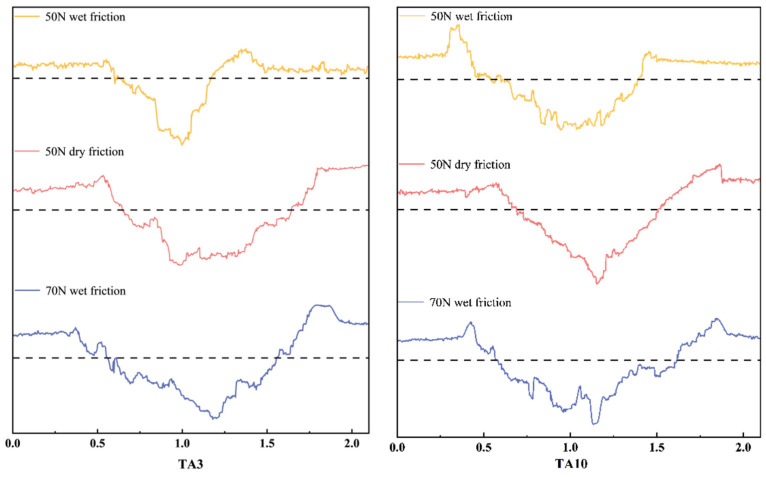
Section profile of friction mark.

To further observe the micro-morphology of the friction marks and accurately measure the average width and depth of the samples, the friction marks were observed by laser confocal microscope and measured with the attached ruler. The friction marks and cross-sectional contours of the friction marks were observed by laser confocal microscope, as shown in Figure 8 and Figure 9, respectively. It can be seen that the extrusion of the friction ball causes the plastic deformation of the sample, and there are a large number of bumps on both sides of the friction marks. At the same time, it can be seen that there are many gullies in the friction trace along the sliding direction of the friction. Among them, the friction mark width of TA3 under the 70 N wet friction condition is the largest, and that of the TA3 under the 50 N wet friction condition is the smallest.

**Figure 10:**
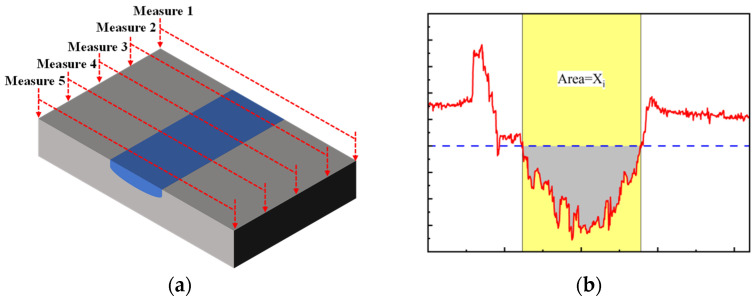
Measurement of average wear width, average wear depth and profile area. (**a**) Measurement method of wear scar width and depth, (**b**) The cross-sectional area of the contour is measured by integral.

The area of the sample scanned by laser confocal microscope is 2.1 mm × 0.8 mm, and the width and depth of the cross-section are measured every 0.2 mm (Figure 10a). Therefore, each sample is measured five times, and the error of the measurement result of five times is calculated by the Formula (1).

(1) $$\delta =\sqrt{\frac{\sum_{i=1}^{n}{\left(x_{i}-\bar{x}\right)}^{2}}{n-1}}$$

**Figure 11:**
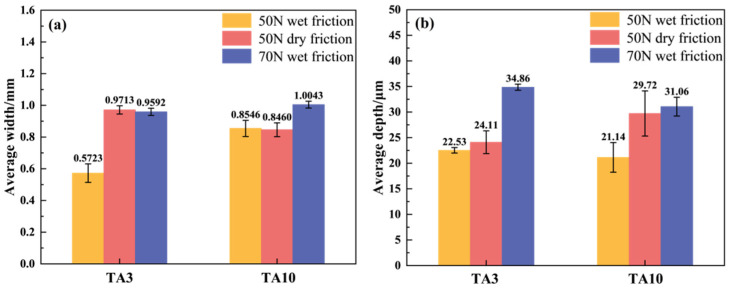
Average wear width and average wear depth ((**a**): average wear width; (**b**): average wear depth).

The average width and depth of the friction marks of TA3 and TA10 specimens under different friction tests are shown in Figure 11. The order of the average width of the friction mark of the sample is as follows: 6# > 2# > 3# > 4# > 5# > 1#, the average width of the friction mark of the TA10 (6#) sample is the largest under the wet friction condition of 70 N, and that of the TA3 (1#) sample is the smallest under the wet friction condition of 1.0043 mm and 50 N, which is only 0.5723 mm. The order of the average depth of the friction marks is as follows: 3# > 6# > 5# > 2# > 1# > 4#, the average depth of the friction marks of TA3 (3#) samples is the largest under 70 N wet friction conditions, at 34.86 μm, and that of TA10 (4#) samples under 50 N wet friction conditions is the smallest, at only 21.14 μm.

In order to compare the wear amount of samples under different friction conditions more accurately, the wear rate can be calculated using the wear volume. The wear volume can be estimated by the area calculated by the integral of the contour curve (Figure 10b), as shown in Formula (2):

(2) $$W_{V}=X_{i}\cdot L$$

where *W_v_* is the wear volume of the sample, mm^3^; *X_i_* is the outline area of friction marks, mm^2^; *L* is the length of friction marks, and *L* = 10 mm is taken in the test. At present, the specific wear rate is commonly used as an important index to measure the friction and wear properties of materials. The formula for the specific wear rate is shown in Formula (3) [38,39]:

(3) $$K=\frac{W_{V}}{PS}$$

In the formula, *K* is the specific wear rate, mm^3^/(N·m), *P* is the friction load, S is the friction sliding distance, m.

**Figure 12:**
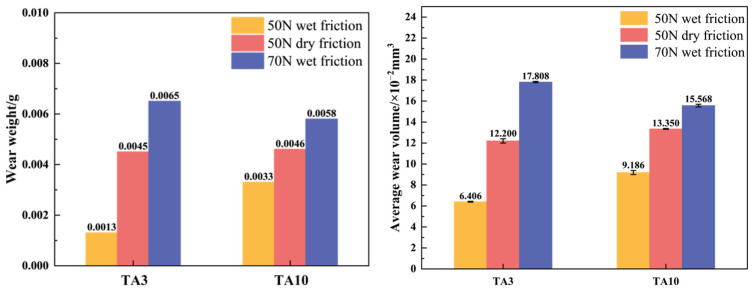
Wear mass and volume of samples.

According to the calculation, the wear volume and specific wear rate of the samples under different working conditions are shown in Table 5, and the order of the specific wear rate of the samples is 5# > 3# > 2# > 6# > 4# > 1#. As mentioned earlier, because some of the materials are not completely detached from the surface, the wear amount of the metal is smaller than the theoretical wear (Figure 12). Therefore, there is a certain difference between the order of the wear volume and the order of the specific wear rate. Under the wet friction condition of 50 N load, the specific wear rates of TA3 and TA10 are 10.677 × 10^−5^ mm^3^/(N·m) and 15.310 × 10^−5^ mm^3^/(N·m), respectively, indicating that TA3 has better wear resistance. The same rule can be obtained by comparing the specific wear rates of TA3 and TA10 under the dry friction condition of 50 N load. However, under the wet friction condition of 70 N load, the specific wear rate of TA3 is 13% higher than that of TA10, and TA10 has better wear resistance. This is consistent with the result that the average friction coefficient of the TA3 (3#) sample is slightly higher than that of the TA10 (6#) sample under the 70 N wet friction condition.

**Figure 13:**
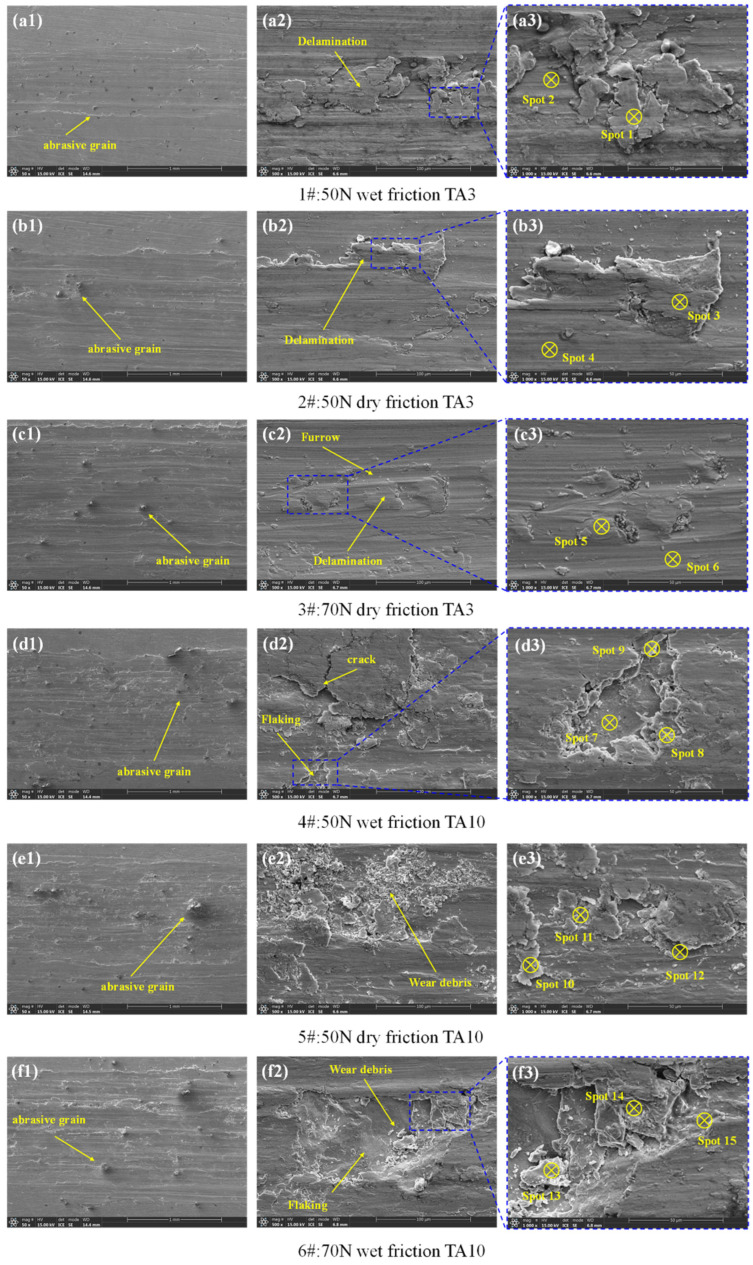
SEM images of wear topography under different friction conditions. (**a1**–**a3**) TA3 during 50 N wet friction, (**b1**–**b3**) TA3 during 50 N dry friction, (**c1**–**c3**) TA3 during 70 N dry friction, (**d1**–**d3**) TA10 during 50 N wet friction, (**e1**–**e3**) TA10 during 50 N dry friction, (**f1**–**f3**) TA10 during 70 N dry friction.

In order to study the friction and wear mechanism under different friction tests, the morphology of surface wear marks was analyzed using a scanning electron microscope, as shown in Figure 13. From the low magnification mirror of Figure 13(a1–f1), it can be seen that abrasive wear occurs in all friction tests, a large number of abrasive particles adhere to the wear mark surface, and the plowing phenomenon is obvious. It can be seen from Figure 13(a2–c2) that the TA3 samples show different degrees of delamination and a small amount of wear debris under three working conditions. Figure 13(d2,f2) shows that the matrix material falls off on the surface of the TA10 (4#) sample under the 50 N wet friction condition and the TA10 (6#) sample under the 70 N wet friction condition, in which the shedding of the sample is the most serious under wet friction test, and the shedding pit exposes the metal matrix and distributes some wear debris. It can be seen from Figure 13(d2–f2) that different degrees of debris accumulation were observed in TA10 samples under three working conditions: the debris particles on the surface of the TA10 (5#) samples were the densest under 50 N dry friction conditions, and micro-cracks were observed under 70 N wet friction conditions. All of these show obvious characteristics of adhesive wear.

Because of the high activity of titanium, titanium and titanium alloys easily form oxide films with high hardness and brittleness on the surface in air or solution, and they easily fall off under extrusion. Therefore, throughout the friction and wear test, the first contact friction with the friction ball is the outermost oxide film. When the oxide film is destroyed, new oxide films continue to form on the exposed surface, and oxidation wear continues to occur. At the same time, the untimely discharge of exfoliated debris will lead to abrasive wear. Before the friction test, the EDS analysis of the surface of TA3 and TA10 demonstrated that the content of the O element on the surface of TA3 was about 12% and the content of the O element on the surface of TA3 was about 14%. In order to further explore the element composition changes of the friction marks after the test, the friction and wear morphologies of the samples under different friction tests were analyzed by EDS. The position of the measuring point is shown in Figure 13(a3–f3), and the EDS results are shown in Table 6. The maximum O content of the grooves on the matrix surface (point 2, point 4 and point 6) is 8.31%. On the other hand, the extrusion of the friction ball leads to the plastic deformation and delamination of the surface oxide film, so the O content in the delamination area (point 1 and point 3) is higher, at 13.02 to 15.67%. Due to the surface tearing and falling off, the edge of the exposed pit (point 8 and point 14) was oxidized in a large area, resulting in a sharp increase in O content, up to 20.49%. The natural oxidation film on the surface of titanium and titanium alloy at room temperature is only less than 20 μm. The measurement of the average depth of the friction marks shows that the thickness of the oxide film is much smaller than the damage depth of the friction surface. Therefore, the samples that underwent different friction tests may have slight oxidation wear. The results show that the load and the oxygen content of the friction medium will affect the oxidation wear. When the load exceeds a certain critical value, the amount of wear increases sharply with the increase in load, which changes from oxidation wear to adhesive wear.

### 3.3. Erosion Wear Performance

In the process of erosion and wear, the surface of the material will be continuously affected by the impact of particles, and the material will be deformed or even removed, which will cause wear and material quality loss. The erosion rate of the sample is calculated using the following Formula (4).

(4) $$E_{v}=\frac{m_{1}-m_{2}}{t}$$

In the formula: *E_v_* is the erosion rate, g/s; *m*_1_ indicates the mass of the specimen before testing, g; *m*_2_ indicates the mass of the specimen after testing, g; *t* is the test time, s.

**Figure 14:**
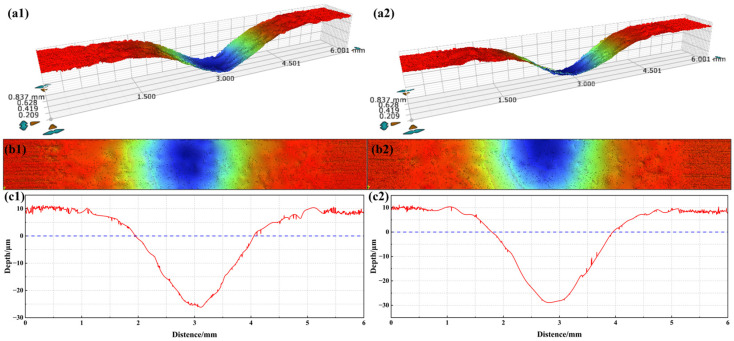
3D surface profile of eroded samples ((**a1**–**c1**): TA3; (**a2**–**c2**): TA10).

Each group of samples for TA3 and TA10 were tested three times, and then the average value was taken. The specific test data are shown in Table 7. From the comparative analysis of the data, we can see that under the same erosion simulation conditions, the erosion rates of TA3 and TA10 are almost the same, and their erosion resistance is similar. To further compare the depth of the erosion pit, typical samples were selected to observe the central area of the erosion pit using a laser confocal microscope. The three-dimensional profile of the erosion pit is shown in Figure 14, and the maximum depths of the observed central area are 28.358 μm and 29.661 μm, respectively.

**Figure 15:**
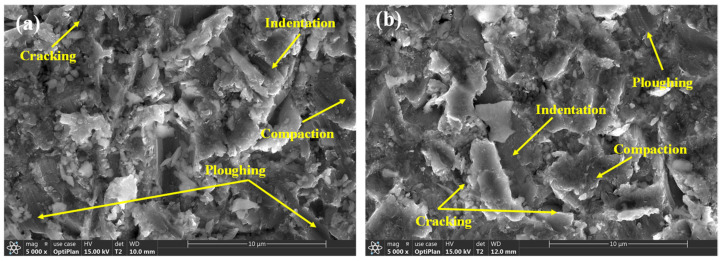
Erosion micromorphology of TA3 and TA10 ((**a**): TA3; (**b**): TA10).

At the impact angle of 45° and the particle impact velocity of 20 m/s, the micro-erosion morphology of TA3 and TA10 are shown in Figure 15. Through the erosion test of 20 min, it can be seen from Figure 15 that even if the hardness and element composition of the two materials are different, the mechanisms of material removal are not different, including the plowing, indentation and cracking mechanisms, which belong to the damage form of typical plastic metal materials impacted by hard particles.

It is generally believed that the spherical smooth abrasive is mainly plowed, while the polygonal abrasive is mainly cut. When the impact angle is 45°, the specimen is subjected to both the shear stress along the surface and the compressive stress on the vertical surface. According to the force analysis, the effect of the shear stress is similar to that of the compressive stress. The shear stress of the sand cuts the surface, as shown in Figure 15, which produces a large number of scratches and furrows. The scratches on the surface along the cutting direction accumulate locally and form a staggered area through the repeated impact of the particles. The materials in the staggered area are easy to tear off from the surface because of the greatest cutting effect. The surface is impacted by the compressive stress of the sand particles, as shown in Figure 15, which produces a large number of massive compactions and pits, with an obvious uplift at the edge of the pits and many irregular cube clastic particles.

## 4. Discussion

**Figure 16:**
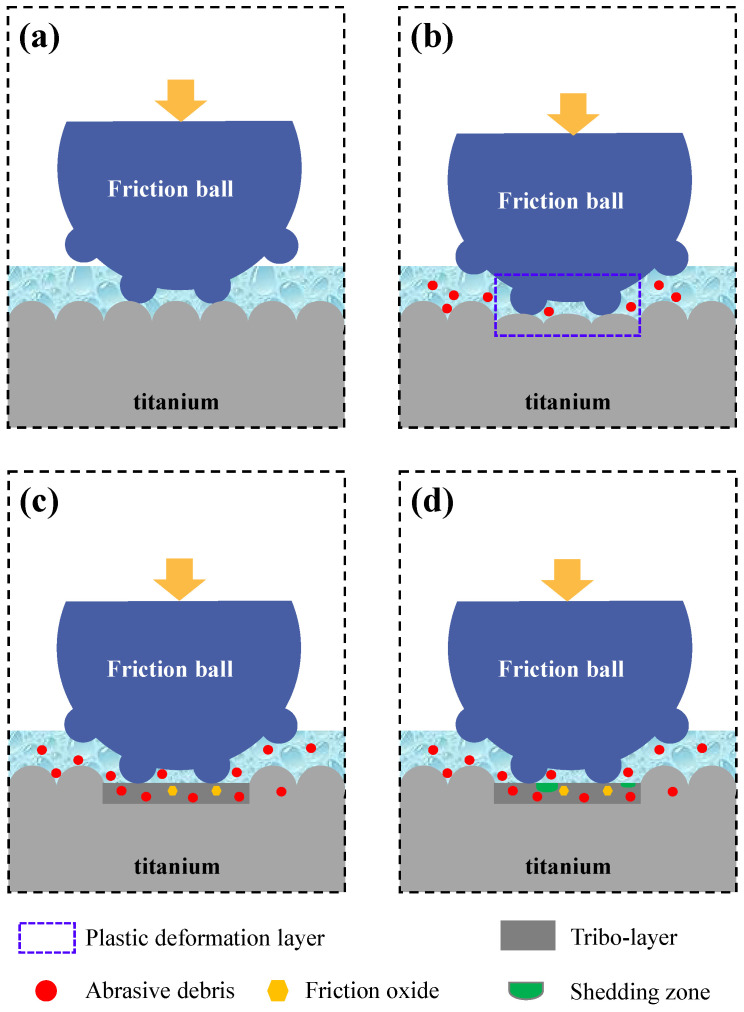
Formation and evolution of friction layer in friction and wear. (**a**) contact phase, (**b**) wear debris generation, (**c**) friction oxide formation, (**d**) generation of shedding zone.

The study shows that the wear process of [40,41] the metal is more complex than that shown in Figure 6. The friction and wear process of titanium and titanium alloy is shown in Figure 16. When the friction begins (Figure 16a), the sample and the friction ball begin to contact the micro-convex body on the contact surface under the action of friction load and friction force. At this time, the plastic deformation or even the fracture of the micro-convex body occurs because the stress exceeds the yield limit of the material, resulting in wear debris (Figure 16b). The elemental analysis in Table 6 shows that the composition of wear debris basically comes from titanium and titanium alloy samples. Some of the wear debris will leave the surface of the friction pair and break away from the friction system, while the other part will stay between the friction pairs and further participate in the wear process. As the sliding process progresses, this debris will be further deformed and broken into smaller particles. Under the action of friction load and friction force, these fine particles will gradually gather and put pressure on the worn surface [42,43,44], forming a friction layer [45].

With the generation of heat in the friction process, friction oxides are formed (Figure 16). At this time, the friction layer can obviously improve the wear performance of the material and reduce the wear rate [46]. With the change in the friction process or friction conditions, the friction layer will tear, collapse and form shedding pits, affecting the wear rate. In the experiment, the sample is placed horizontally, and the wear debris produced in the wear process can be retained on the surface of the sample, which creates conditions for the formation of oxides in the friction layer. However, with different sample materials, the friction load and friction form, the thickness, distribution and properties of the friction layer and the content of oxide in the friction layer are different. It is reported that [47] the type of friction layer is the key factor affecting the wear behavior and mechanism [48]. However, the study shows that the thickness of the friction layer formed under different working conditions has no obvious rule, and the thickness of the friction layer seems to have little effect on the wear behavior of the titanium alloy. Therefore, we are mainly concerned about the integrity of the friction layer and the oxide content.

Under the condition of 50 N wet friction, at the initial stage of wear, the surface of the TA10 (4#) sample is torn into pits and the material collapses due to adhesive wear, which affects the integrity of the friction layer, and has a high wear rate. However, under the wet friction condition of 50 N, the TA3 (1#) sample mainly causes abrasive wear, and the surface is relatively intact, so TA3 has a lower specific wear rate and better wear resistance than TA10. Under the condition of wet friction, when the load increases to 70 N and the surface of the TA10 (6#) sample is further deformed and tearing, a large amount of wear debris is produced, and some of the wear debris is compacted into a surface friction layer while acting as a solid lubricant. On the other hand, however, under the 70 N wet friction condition, the surface hardness of the TA3 (3#) sample is lower, and the depth of the friction ball is larger, which leads to a larger cross-sectional area of friction marks, larger wear volume and higher wear rate. Therefore, when the friction load is larger, the wear resistance of TA10 is stronger. It is speculated that the difference in the friction process may be due to the differences in hardness, microstructure and chemical composition between TA3 and TA10. At the same time, it also means that in practical engineering applications, the TA10 is the preferable pipeline material in the face of the harsh working conditions of the friction load.

Early studies have shown that the erosion depth and erosion mechanism can be influenced by sand particle size, velocity, impact angle, the material property and the hardness ratio between the eroding particles and eroded material. It can be seen that the test conditions are the main influencing factors of the erosion rate. Because the surface hardness difference between TA3 and TA10 is small, the erosion rate of TA10 is slightly larger than that of TA3. At the same time, TA3 and TA10 are plastic materials, so the erosion mechanism is similar. So, TA3 and TA10 have the same status when selecting materials based on erosion resistance. Controlling the sand content, sand shape and size, the gas flow rate and impact angle in the pipeline are effective means to reduce pipeline damage.

## 5. Conclusions

The friction and wear tests of TA3 and TA10 were carried out based on different friction loads and friction forms. The gas–solid erosion tests of TA3 and TA10 were carried out under simulated working conditions. The friction and wear properties and gas–solid erosion wear properties of TA3 and TA10 were analyzed. The following conclusions can be drawn:

(1) The friction load and friction form have a certain influence on the wear process of TA3 and TA10. Under the wet friction condition of 70 N friction load, the friction coefficient and wear amount of TA3 are the highest at 0.44 and 0.0065 g, respectively. When the friction load is 50 N, whether under dry friction or wet friction, TA3 shows better wear resistance; when the friction load is 70 N, the wear resistance of TA10 is stronger. TA10 is a suitable pipe material under the condition of bad friction load.
(2) In the friction and wear test, the wear forms of TA3 and TA10 include abrasive wear, adhesive wear and oxidation wear. Under the wet friction form of the 70 N friction load, the wear form of the TA3 sample is mainly abrasive wear, while the surface of the TA10 sample appears torn and shedding and the main wear form is adhesive wear.
(3) Through the comparative analysis of the erosion micro-morphology of the samples, it was found that under the simulated working conditions with a 45° impact angle, the erosion rates of TA3 and TA10 were 1.01 × 10^−3^ g/s and 0.94 × 10^−3^ g/s. The erosion mechanism is the same, including plowing, indentation and cracking.

## Figures and Tables

**Table 1:** Chemical composition of TA3 and TA10.

Material	Fe	C	N	H	O	Ni	Mo	Ti
TA3	0.35	0.08	0.03	0.01	0.25	-	-	Bal.
TA10	0.25	0.06	0.01	0.01	0.2	0.75	0.32	Bal.

**Table 2:** Hardness test results of TA3 and TA10.

Material	Test 2	Test 3	Test 4	Test 5	Test 6	Average
TA3	148.23	146.55	150.21	149.88	148.37	148.65
TA10	198.52	199.83	201.54	203.62	201.78	201.06

**Table 3:** Friction and wear test scheme.

Test Number	Material	Load/N	Friction Form	Time/s	Stroke/mm	Speed/ms^−1^
1#	TA3	50	wet friction	1200	10	10
2#	TA3	50	dry friction	1200	10	10
3#	TA3	70	wet friction	1200	10	10
4#	TA10	50	wet friction	1200	10	10
5#	TA10	50	dry friction	1200	10	10
6#	TA10	70	wet friction	1200	10	10

**Table 4:** Parameter settings for gas–solid erosion test.

Material	Impact Angle	Particle Impact Velocity (m/s)	Particle Mass Flow Rate (g/min)	Time (min)
TA3	45°	20	1.55	20
TA10	45°	20	1.55	20

**Table 5:** Wear volume and specific wear rate of samples under different working conditions.

Test Serial Number	Wear Volume *W_v_* (10^−2^ mm^3^)	Specific Wear Rate *K* (10^−5^ mm^3^·N^−1^·m^−1^)
1#	6.406	10.677
2#	12.200	20.333
3#	17.808	21.200
4#	9.186	15.310
5#	13.350	22.250
6#	15.568	18.533

**Table 6:** Element composition according to EDS analysis from Figure 13.

Test Serial Number	Spot	Ti	Fe	O	Cl	N	Na
1#	1	82	0.71	15.67	0.05	1.21	0.36
	2	89.34	0.16	7.88	0.17	1.32	1.13
2#	3	82.35	0.47	13.02	0.48	3.40	0.28
	4	86.31	0.12	8.31	0.21	4.85	0.21
3#	5	87.54	0.08	8.50	0.14	3.70	0.04
	6	88.98	-	7.00	-	4.00	0.02
4#	7	80.87	0.25	13.91	0.17	3.56	0.18
	8	63.46	12.48	20.49	0.59	0.43	0.39
	9	63.35	2.01	9.98	8.81	4.01	10.89
5#	10	77.58	0.86	15.39	0.44	4.29	0.85
	11	72.63	4.22	17.45	1.33	2.06	1.13
	12	79.14	2.36	13.70	0.16	3.32	0.39
6#	13	80.58	0.47	13.89	0.54	3.32	0.24
	14	73.88	0.47	20.04	0.96	2.90	0.85
	15	69.92	0.86	8.95	7.52	2.86	9.03

**Table 7:** TA3 and TA10 erosion test data.

Material	Time t/s	Pre-Test Quality *m*_1_ (g)	Post-Test Quality *m*_2_ (g)	Erosion Rate *E_v_* (g/s)	Average Erosion Rate *E_v_* (g/s)
TA3	1200	14.4868	13.2748	1.01 × 10^−3^	1.01 × 10^−3^
		14.3547	13.1549	0.998 × 10^−3^	
		14.5542	13.3191	1.03 × 10^−3^	
TA10		13.7539	12.7028	0.876 × 10^−3^	0.940 × 10^−3^
		13.5572	12.4211	0.947 × 10^−3^	
		13.4885	12.2915	0.998 × 10^−3^	

## Data Availability

Data is unavailable due to privacy restrictions.
